# Quality care

**DOI:** 10.1186/1758-5996-7-S1-A192

**Published:** 2015-11-11

**Authors:** Ilse Deutsch, Laura Pardo Muñagorri

**Affiliations:** 1 Hospital Pereira Rossell, Uruguai

## Background

Diabetes mellitus is one of the most common chronic diseases in childhood and adolescence, with increasing incidence. The main focus of its management is to achieve quality care by a multidisciplinary team who also deal with the education of the child and their family. Improvements in the care process usually precede improvements in metabolic control of patients. At its base are the identification, recording and analysis of health care quality markers.

## Objective

To evaluate the quality of the care process of the Diabetes Unit at the Pereira Rossell Hospital Center.

## Materials and methods

A descriptive, retrospective study, using clinical records of patients monitored at DU between 1/7/2012 and 1/7/2013. The criteria for good quality care were: good nutritional status, adequate pubertal development, lack of hospitalizations, adequate frequency of clinical controls, preventive controls of micro and macrovascular disease, normal Results: Of the preventive controls, application of glycosylated hemoglobin test and appropriate value. The quality of the care would be considered good to the presence of 7 met criteria, acceptable to the presence of 6, and poor below 6. Absolute frequencies and percentages were used. Results: 83 patients were included, 37 (45%) presented good quality care, 20 (24%) acceptable and 26 (31%) bad. The two main flaws were in the average values of glycosylated hemoglobin and in the number of annual tests requested.

## Conclusions

With regular assessment of the quality of care strengths can be detected and weaknesses can be corrected. After five yrs. of operation, the Diabetes Unit (DU) got almost half of patients received good quality care in this disease. However, monitoring compliance with controls should be improved by strengthening the socio-economic support and education.

